# Association between sleep disorders and cognitive dysfunctions in non-demented patients with advanced Parkinson’s disease

**DOI:** 10.1007/s00415-021-10726-z

**Published:** 2021-07-30

**Authors:** Elisa Montanaro, Alberto Romagnolo, Margherita Fabbri, Carlo Alberto Artusi, Gabriele Imbalzano, Mario Giorgio Rizzone, Leonardo Lopiano, Maurizio Zibetti

**Affiliations:** 1 Department of Neuroscience “Rita Levi Montalcini”, University of Turin, Via Cherasco 15, 10126 Turin, Italy; 2 Neurology 2 Unit, A.O.U. Città della Salute e della Scienza di Torino, Corso Bramante 88, 10126 Torino, Italy; 3 Department of Neurosciences, Clinical Investigation Center CIC 1436, Parkinson Toulouse Expert Center, NS-Park/FCRIN Network and NeuroToul COEN Center, Toulouse University Hospital, INSERM, University of Toulouse 3, Toulouse, France

**Keywords:** Parkinson’s disease, Non-motor symptoms, Sleep disorders, Cognitive impairment

## Abstract

**Background:**

Parkinson’s disease **(**PD) is increasingly recognized as a multidimensional disorder, characterized by several non-motor symptoms, including disturbances of sleep and cognition. Current studies on the relationship between sleep problems and neuropsychological functions, mainly conducted in early to moderate PD patients, outline mixed results. In this study, we analysed the relationship between subjectively reported sleep alterations and cognitive functions in a large cohort of 181 advanced PD patients.

**Methods:**

All consecutive, non-demented, advanced PD patients candidates for device-aided therapy completed two self-administered sleep questionnaires—the Parkinson’s Disease Sleep Scale (PDSS-2) and the Epworth Sleepiness Scale (ESS)—and underwent a comprehensive battery of neuropsychological tests encompassing five cognitive domains (reasoning, memory, attention, frontal executive functions, and language).

**Results:**

Patients showed mild to moderate sleep problems (PDSS-2 score: 23.4 ± 1.2) and mild daytime sleepiness (ESS 8.6 ± 5.1). A significant correlation was found between PDSS-2 total score and non-verbal reasoning, as well as attentive skills, executive functions, and language abilities. No correlations were found between sleep measures and memory tests scores. Patients with clinically relevant sleep disturbances performed worse on attention, executive functions, and language. No significant correlations were found between daytime sleepiness and any neuropsychological test.

**Conclusions:**

In advanced PD patients, sleep disturbances selectively correlate with specific neuropsychological functions and not with short-term memory and consolidation. Even if confirmations by means of longitudinal studies are needed, our observations suggest the importance of considering treatment of sleep disturbances to minimize their potential impact on cognition.

## Introduction

Parkinson’s disease (PD) is a progressive neurodegenerative disorder, traditionally defined by its cardinal motor symptoms. However, during the last decades, PD has been identified as a multidimensional disorder characterized by several non-motor symptoms, including sleep disturbances and cognitive alterations. Sleep problems are known to contribute to neuropsychological deficits in otherwise healthy people [1]. The association between sleep problems and cognitive disorders has been explored in neurodegenerative disease, such as Alzheimer’s disease (AD) [2–4] and PD [5–10].

Both cognitive dysfunctions and sleep problems represent a heterogeneous group of neurological symptoms observed in PD patients with a great variability in presentation and time of onset [11]. Sleep problems, encompassing insomnia, vivid dreams, restless legs syndrome, rapid eye movement sleep behavior disorder (RBD), and excessive daytime sleepiness (EDS), are very common in PD patients, affecting up to 98% of subjects, with increasing prevalence as the disease progresses [11].

Studies investigating the relationship between sleep disturbances and neuropsychological functions, conducted mainly in early to moderate PD patients, support the notion that some sleep alterations like RBD and EDS are connected to the development of cognitive dysfunctions and dementia in PD patients [5–10, 12].

To support and better characterize the association between sleep disturbances and neuropsychological functions in advanced PD, we performed a retrospective data analysis of a large cohort of candidates for device-aided therapies, evaluated by a comprehensive neuropsychological battery and validated sleep scales.

## Methods

### Participants

In this retrospective study, we included all consecutive advanced PD patients who were candidates for device-aided therapies to the Movement Disorder Center of the Turin University Hospital. All patients had a diagnosis of idiopathic PD, as per the Movement Disorders Society criteria [13]. Advanced PD was defined as the persistence of motor fluctuations and/or troublesome dyskinesia limiting the activities of daily living in spite of repeated adjustments of medication [14]. To guarantee a reliable evaluation of sleep disturbances, and to avoid measurements affected by a too severe impairment of cognitive functions or by an important psychiatric disorder, we included only patients with a Mini-Mental State Examination (MMSE) [15] score ≥ 24, and without major neurocognitive disorder or major depression according to DSM 5 criteria. The Local Ethical Committee approved the study protocol (Protocol number: 0025346 of 11 May 2016; Comitato Etico Interaziendale A.O.U. Città della Salute e della Scienza di Torino—A.O. Ordine Mauriziano di Torino—A.S.L. TO1), and each patient signed a written informed consent to participate in the study.

### Clinical assessments

#### Neurological examination

All patients were characterized according to the Unified Parkinson’s Disease Rating Scale (UPDRS) [16]. The UPDRS parts II and III, the Hoehn and Yahr (HY) stage, and the Schwab and England scale of activities of daily living were scored both in the “On” and “Off” state. Levodopa equivalent daily dose (LEDD) was calculated as per a validated conversion table [17]. Disease duration was calculated from age at diagnosis.

#### Sleep related measures

Sleep quality was assessed by means of the modified version of the Parkinson’s Disease Sleep Scale (PDSS-2) [18, 19], a self-administered questionnaire encompassing 15 questions evaluating several aspects of sleep disturbances. Each question refers to the previous week and is rated through a categorical scale describing the frequency of the disturbance (0 = never; 1 = occasionally; 2 = sometimes; 3 = often; 4 = very often). Beside general sleep quality (item 1), questions encompass difficulties in falling (item 2) or staying asleep (item 3), nocturnal restless legs syndrome (items 4 and 5), vivid distressing dreaming (item 6), hallucinations (item 7), nocturnal urinary urgency (item 8), immobility at night (item 9), pain (item 10), muscle cramps (item 11), painful posturing in the morning (item 12), tremor on waking (item 13), lack of repose from sleep (item 14) and sleep apnea (item 15). An overall sum score, ranging from 0 (no disturbance) to 60 (maximum nocturnal disturbance), provides a general evaluation of sleep quality; score ≥ 18 is indicative of clinically relevant PD-related sleep disturbances [19]. Moreover, three domains are calculated by summing individual item scores in groups of five, with a maximum score of 20: “Disturbed sleep” (DS; items 1–3, 8 and 14): “Motor symptoms at night” (MSN; items 4–6, 12 and 13); “PD symptoms at night” (PDSN; items 7, 9–11 and 15) [18, 20].

Moreover, patients were asked to complete the Epworth Sleepiness Scale (ESS) [21], to assess daytime sleepiness. This scale evaluates the chance of “dozing” (0 = would never doze; 1 = slight chance of dozing; 2 = moderate chance of dozing; 3 = high chance of dozing) in eight daily situations: sitting and reading (item 1); watching TV (item 2); sitting, inactive in a public place (e.g. a theatre or a meeting) (item 3); as a passenger in a car for an hour without a break (item 4); lying down to rest in the afternoon when circumstances permit (item 5); sitting and talking to someone (item 6); sitting quietly after a lunch without alcohol (item 7); in a car, while stopped for a few minutes in the traffic (item 8). Scores range from 0 to 24; scores ≥ 10 are indicative of excessive daytime sleepiness.

#### Cognitive and behavioral measures

A comprehensive neuropsychological battery [22, 23] was administered to evaluate five cognitive domains: reasoning [Raven Color Progressive Matrices Test (RCPMT)] [24]; memory [Corsi’s Block Tapping Test (CBT) and Paired Associate Learning (PAL)] [25, 26]; attention [Digit Cancellation Test (DCT), Trail Making Test A (TMA)] [25, 27]; frontal executive functions [Trail Making Test B (TMB), Frontal Assessment Battery (FAB)] [27, 28]; and language skills [phonemic (PVF) and category verbal fluency (CVF)] [25, 29]. Higher scores in RCPMT, CBT, PAL, DCT, FAB, PVF, CVF indicate better performance; higher scores in TMA, TMB indicate worse performance. Depressive and apathetic symptoms were assessed by means of the Beck Depression Inventory II (BDI) [30] and the Marin Apathy Scale (MAS) [31], respectively.

All neuropsychological assessments were performed in the best clinical condition (“On” condition).

### Statistical analyses

Descriptive statistics were summarized as mean ± standard deviation. A linear regression analysis was used to evaluate the association between PDSS-2 scores, the three PDSS-2 domains scores, and ESS scores (independent variables) and cognitive tests scores (dependent variables), adjusted for age, disease duration, LEDD, and years of education. Analysis of covariance (ANCOVA) was used to compare cognitive tests scores (dependent variables) of patients with or without significant sleep alterations (i.e. PDSS-2 ≥ 18; ESS ≥ 10), adjusted for age, disease duration, LEDD, and years of education (covariates); ANCOVA assumption of homogeneity of regression slopes was verified. Age, disease duration, years of education, and LEDD were chosen as covariates on the basis of their well-proven influence on cognition and sleep performances [32–34]. The relationship between cognitive measures and PDSS-2 or ESS scores was considered as primary outcomes, while the analyses concerning the PDSS-2 subscores were considered as exploratory outcomes. All *p* values reported are two-tailed and a *p* < 0.05 was considered statistically significant. Data were analysed using the Statistical Package for the Social Sciences (SPSS 26 for Windows, Chicago, IL).

## Results

Data from a total of 195 consecutive PD patients were analysed. Nine patients were excluded for a MMSE < 24, three for the presence of major depression and two for missing data. A total of 181 PD patients were included in the study. The demographic and clinical features of included participants are reported in Table 1. Overall, the mean PDSS-2 and ESS scores were 23.4 ± 11.2 and 8.6 ± 5.1, respectively. As per inclusion criteria, the mean MMSE score was within normal ranges (28.6 ± 1.6); mean depressive and apathetic symptoms were mild (BDI-II = 12.4 ± 7.5; MAS = 12.2 ± 6.1) (Table 1).

**Table 1:** Demographic and clinical variables

Age, years	61.1 ± 8.3 (35–77)
Education, years	10.1 ± 4.2 (3–23)
Men–women	114–67 (63–37%)
Disease duration, years	11.7 ± 4 (4–32)
UPDRS I	2.4 ± 2.2 (0–16)
UPDRS II on	9.8 ± 7.3 (0–36)
UPDRS II off	20.1 ± 9.5 (1–75)
UPDRS III on	15.1 ± 8.7 (1–45.5)
UPDRS III off	38.6 ± 13.7 (8–83)
UPDRS IV	6.6 ± 3.7 (0–17)
Hoehn and Yahr on	2.1 ± 0.7 (0–4)
Hoehn and Yahr off	3 ± 1 (0–5)
Schwab and England on	89.5 ± 12 (50–100)
Schwab and England off	60.9 ± 20.1 (10–90)
LEDD, mg	1140.1 ± 447.3 (0–2567.5)
Epworth sleepiness scale	8.6 ± 5.1 (0–23)
Parkinson’s disease sleep scale	23.4 ± 11.2 (2–53)
PDSS-2 disturbed sleep	9.9 ± 3.8 (0–20)
PDSS-2 motor symptoms at night	7.5 ± 4.9 (0–20)
PDSS-2 PD symptoms at night	6.1 ± 4.5 (0–18)
Beck depression inventory	12.4 ± 7.5 (0–34)
Marin apathy scale	12.2 ± 6.1 (0–28)
Mini-Mental State Examination	28.6 ± 1.6 (24–30)
Raven coloured progressive matrices test	28.0 ± 5.8 (0–36)
Corsi’s block tapping test	4.5 ± 0.9 (2–7)
Paired associative learning	12.1 ± 3.2 (5.5–21)
Digit cancellation test	46.6 ± 9.9 (5–60)
Trail making test A	57.3 ± 42.3 (19–350)
Frontal assessment battery	15.4 ± 2.6 (7–18)
Trail making test B	168.1 ± 139.9 (39–600)
Phonemic verbal fluency	38.0 ± 15 (11–92)
Category verbal fluency	22.1 ± 6.2 (8.25–39)

All data are reported as means ± standard deviation (range), or absolute numbers (percentage)

*LEDD* levodopa equivalent daily dose, mg

### Neuropsychological factors associated with sleep disorders

As shown in Table 2, after adjusting for age, diseases duration, LEDD, and years of education, a significant association was observed between higher PDSS-2 scores, indicative of greater sleep disturbances, and worse performances in tests assessing reasoning (RCPTM: *ß* = − 0.214; *p* = 0.002), attention (DCT: *ß* = − 0.194; *p* = 0.007), executive functions (TMB: *ß* = 0.156; *p* = 0.031; FAB: *ß* = − 0.139; *p* = 0.049) and category verbal fluency (CVF: *ß* = − 0.167; *p* = 0.027). No significant correlations were found between PDSS-2 scores and memory test scores (CBT: *ß* = − 0.071; *p* = 0.356; PAL: *ß* = − 0.023; *p* = 0.783).

**Table 2:** Correlations between sleep disturbances and cognitive performances

	ESS	PDSS-2 total score	PDSS-2 DS	PDSS-2 MSN	PDSS-2 PDSN
Cognitive screening					
Mini-Mental State Examination	0.043	− 0.173*	− 0.022	− 0.164*	− 0.188*
Reasoning					
Raven coloured progressive matrices test	0.014	− 0.214*	− 0.078	− 0.200*	− 0.214*
Memory					
Corsi’s block tapping test	0.003	− 0.071	− 0.005	− 0.112	− 0.144
Paired associative learning	0.058	− 0.023	0.039	− 0.109	− 0.005
Attention					
Digit cancellation test	0.050	− 0.194*	− 0.099	− 0.202*	− 0.175*
Trail making test A	− 0.084	0.076	0.102	0.074	0.062
Executive functions					
Trail making test B	0.015	0.156*	0.092	0.164*	0.142*
Frontal assessment battery	− 0.040	− 0.139*	0.054	− 0.153*	− 0.131
Language					
Phonemic verbal fluency	0.057	− 0.118	− 0.045	− 0.126	− 0.138
Category verbal fluency	0.056	− 0.167*	− 0.125	− 0.216*	− 0.105

Values represent the correlation coefficient (*ß*). Linear regression analysis was adjusted for age, disease duration, LEDD, and years of education

*ESS* Epworth sleepiness scale, *PDSS-2* Parkinson’s disease sleep scale. PDSS-2 domains: *DS* disturbed sleep, *MSN* motor symptoms at night, *PDSN* PD symptoms at night

*Significant correlation (*p* < 0.05)

Considering the three PDSS-2 subscales separately, a significant correlation was found between: (a) “Motor symptoms at night” and reasoning (RCPTM: *ß* = − 0.200; *p* = 0.005), attention (DCT: *ß* = − 0.202; *p* = 0.006), executive functions (TMB: *ß* = 0.164; *p* = 0.026; FAB: *ß* = − 0.153; *p* = 0.047), and language (CVF: *ß* = − 0.216; *p* = 0.005); and (b) “PD symptoms at night” and reasoning (RCPTM: *ß* = − 0.214; *p* = 0.002), attention (DCT: *ß* = − 0.175; *p* = 0.018), and executive functions (TMB: *ß* = 0.142; *p* = 0.049). There were no significant correlations between “Disturbed sleep” subscale and any of the cognitive functions tested (Table 2).

Clinically relevant PD-specific sleep disturbances, denoted by a score ≥ 18 on the PDSS-2 scale, were reported in 59 out of 181 patients (32.6%). These patients were older (59.1 ± 8.5 vs. 62 ± 8.1 years; *p* = 0.036), had lower education (10.7 ± 3.9 vs. 9.8 ± 4.3 years; *p* = 0.077) and similar disease duration (11.8 ± 5.1 vs. 11.7 ± 3.4 years; *p* = 0.467).

**Fig. 1:**
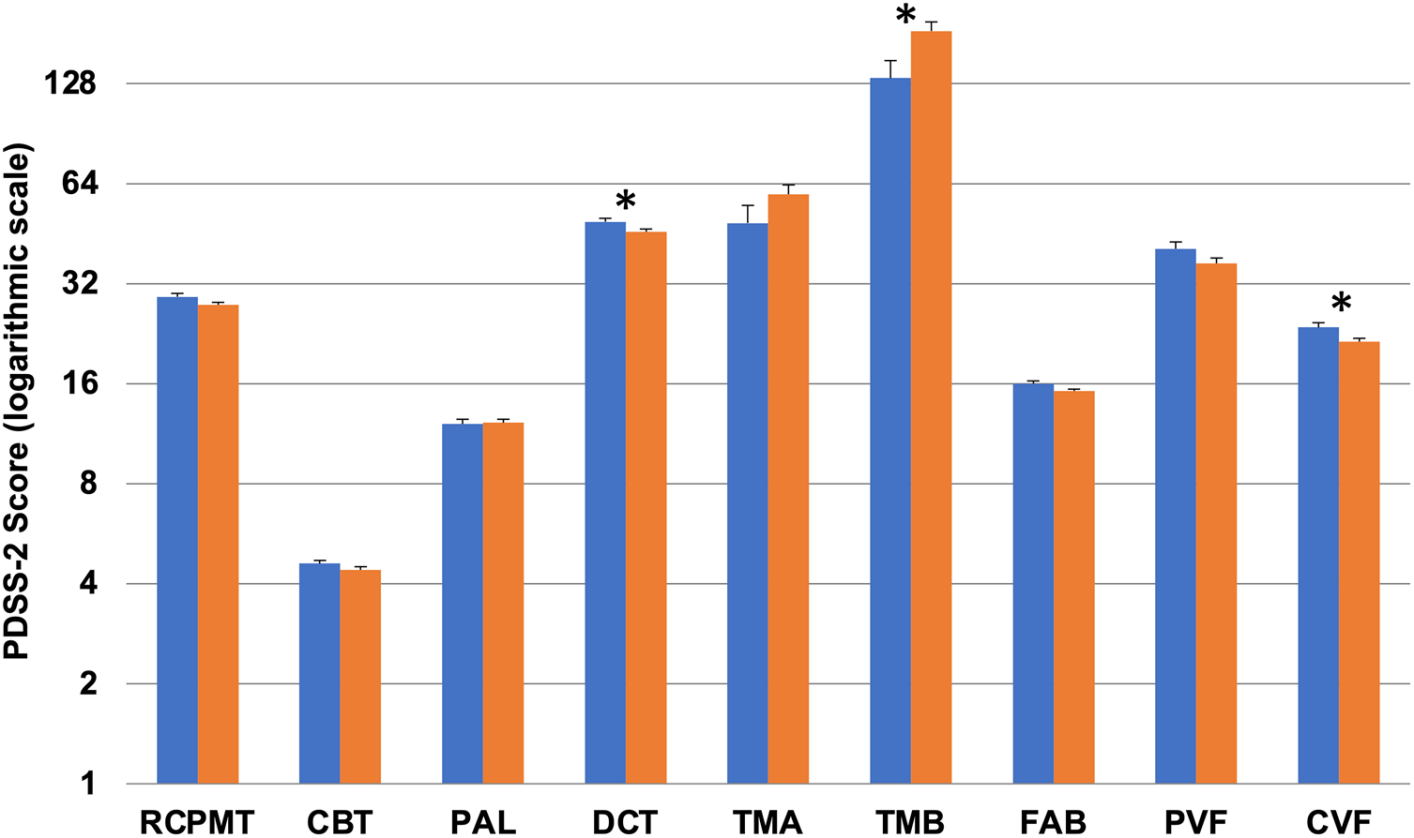
Neuropsychological test scores of patients with and without clinically relevant sleep disturbances at the PDSS-2. Patients with clinically relevant sleep disturbances (PDSS-2 ≥ 18; orange columns) performed worse than patients without sleep disturbances (PDSS-2 < 18; blue columns) on neuropsychological tests assessing attention, executive functions, and language. Values are presented as means and standard errors, adjusted for age, disease duration, LEDD, and years of education (analysis of covariance). PDSS-2 values are presented as a logarithmic scale. *CBT* Corsi’s block tapping test, *CVF* category verbal fluency, *DCT* digit cancellation test, *FAB* frontal assessment battery, *PAL* paired associate learning, *PVF* phonemic verbal fluency, *RCPMT* raven colour progressive matrices test, *TMA* trail making test A, *TMB* trail making test B. *Statistically significant difference (*p* < 0.05)

After adjusting for age, diseases duration, LEDD, and years of education, patients with clinically relevant sleep disturbances presented with similar global cognitive performance than patients without significant sleep alterations (MMSE: 28.8 ± 0.2 vs. 28.5 ± 0.1; *F* = 1.594; *p* = 0.209). However, they performed worse on attention (DCT: 49.1 ± 1.2 vs. 45.8 ± 0.9; *F* = 4.960; *p* = 0.027), executive functions (TMB: 132.8 ± 17.2 vs. 184.1 ± 12; *F* = 5.919; *p* = 0.016), and language (CVF: 23.6 ± 0.8 vs. 21.4 ± 0.5; *F* = 5.225; *p* = 0.024). No significant differences were found in the other neuropsychological tests (Fig. 1).

### Neuropsychological factors associated with daytime sleepiness

As reported in Table 2, no significant correlations were found between ESS scores and cognitive performances.

**Fig. 2:**
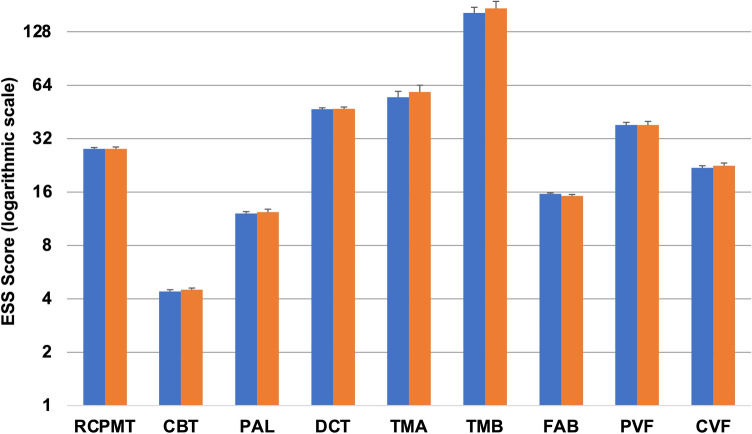
Neuropsychological tests scores of patients with and without clinically relevant daytime sleepiness at the ESS. Patients with (ESS ≥ 10; orange columns) and without (ESS < 10; blue columns) clinically relevant daytime sleepiness showed similar scores in all neuropsychological tests. Values are presented as means and standard errors, adjusted for age, disease duration, LEDD, and years of education (analysis of covariance). ESS values are presented as a logarithmic scale. *CBT* Corsi’s block tapping test, *CVF* category verbal fluency, *DCT* digit cancellation test, *FAB* frontal assessment battery, *PAL* paired associate learning, *PVF* phonemic verbal fluency, *RCPMT* raven colour progressive matrices test, *TMA* trail making test A, *TMB* trail making test B. *Statistically significant difference (*p* < 0.05)

48 out of 181 (26%) showed clinically relevant daytime sleepiness (ESS score ≥ 10). After adjusting for age, diseases duration, LEDD, and years of education, no significant differences were observed between patients with and without excessive daytime sleepiness in all neuropsychological tests (Fig. 2).

## Discussion

We analysed the relationship between sleep disturbances and daytime sleepiness, assessed by means of validated clinical scales (ESS and PDSS-2), and specific cognitive functions in a large sample of advanced PD patients. We found that subjective complaints of sleep disorders, measured by the PDSS-2 scale, correlated with cognitive impairment in several specific cognitive domains. Interestingly, patients with clinically relevant sleep complaints performed worse than those without relevant sleep complaints, in terms of reasoning, attention, executive functions, and verbal fluency, but not memory. Analysing the three main PDSS-2 subscores separately, we found that motor problems at night and PD-specific symptoms at night correlated with neuropsychological dysfunctions in all cognitive domains explored, excluding memory, whereas no correlations were found for disturbed sleep. Finally, no correlations were found between daytime sleepiness and impairment of cognitive functions.

These observations may be of particular interest when planning intervention studies that assess the effects of treatment of sleep disorders on cognition. Further in-depth analysis considering correlations between cognitive functions and single items of the PDSS-2 scale may suggest specific sleep disturbances on which to focus treatment.

Sleep problems have been shown to contribute to neuropsychological deficits both in healthy people and in neurodegenerative disorders, including PD. However, to date, research on the relationship between sleep alterations and cognition in PD still describe a mixed picture. In contrast with our data, excessive daytime sleepiness was described as a significant predictor of slowed processing speed [6] and was correlated with impairment in all cognitive domains, except language, in a cohort of cognitively impaired PD patients [11]. In their meta-analysis, Pushpanathan and colleagues [7] assessed the effect of poor sleep (e.g. insomnia, sleep-related breathing disorder) on cognition in PD showing significant adverse effects of poor sleep in global cognitive functioning, verbal recall, verbal recognition, set shifting, executive updating, generativity, and fluid reasoning. Our data confirm these findings, with the exception of memory impairment. Moreover, our findings are similar to those obtained by Stavitsky and colleagues [9] using actigraphic measures, suggesting an association between poor sleep efficiency and attention/executive cognitive alterations, but not memory.

The screening of PD patients for sleep disorders with validated questionnaires appears to be useful to identify those patients with clinically relevant sleep disorders, possibly associated with a greater degree of cognitive impairment, even in the absence of a polysomnographic assessment, which is not always readily available in clinical practice. Moreover, our data confirm that screening for sleep disorders is also suitable in the context of patient selection for device-aided therapies, given the reduction of sleep alterations after both deep brain stimulation and LCIG infusion [35, 36], with a consequent improvement of patients’ quality of life.

The major strengths of this study are represented by the large sample size and the detailed clinical assessment of neuropsychological functions, whereas the major shortcoming is represented by the lack of instrumental assessment of sleep disorders. Moreover, as per study design, we chose to include only patients without significant cognitive impairment, resulting to a reduced generalizability of our findings. Even though this is an association study, and no causative role can by established, it is plausible to hypothesise that a prompt identification and treatment of sleep disorders has the potential to improve cognition in advanced PD patients. The amelioration of sleep and cognitive alterations could also drive an improvement of the patients’ and caregivers’ quality of life, often negatively affected by these non-motor symptoms [37].

In conclusion, in this cohort of advanced, non-demented, PD patients, we provide evidence of a selective association between subjective sleep disturbances and impaired performance in objective cognitive tests related to specific neuropsychological functions.

Further longitudinal studies are needed to determine whether sleep disorders are risk factors for cognitive decline and dementia in PD, and to understand the underlying mechanisms. Future intervention studies assessing the effects of treatment of sleep disorders on cognition might lead to new opportunities for the prevention of cognitive decline and dementia in PD patients.

## Data Availability

The data that support the findings of this study are available from the corresponding author, upon reasonable request. E. Montanaro and A. Romagnolo have full access to all the data in the study and take responsibility for the integrity of the data, the accuracy of the data analysis, and the conduct of the research.
